# Evidence for encounter-conditional, area-restricted search in a preliminary study of Colombian blowgun hunters

**DOI:** 10.1371/journal.pone.0207633

**Published:** 2018-12-12

**Authors:** Cody T. Ross, Bruce Winterhalder

**Affiliations:** 1 Max Planck Institute for Evolutionary Anthropology, Department of Human Behavior, Ecology and Culture, Leipzig, Germany; 2 University of California Davis, Department of Anthropology, Davis, CA, United States of America; Princeton University, UNITED STATES

## Abstract

Active search for prey is energetically costly, so understanding how foragers optimize search has been central to foraging theory. Some theoretical work has suggested that foragers of randomly distributed prey should search using Lévy flights, while work on area-restricted and intermittent search strategies has demonstrated that foragers can use the information provided by prey encounters to more effectively adapt search direction and velocity. Previous empirical comparisons of these search modes have tended to rely on distribution-level analyses, due to the difficulty of collecting event-level data on encounters linked to the GPS tracks of foragers. Here we use a preliminary event-level data-set (18.7 hours of encounter-annotated focal follows over 6 trips) to show that two Colombian blowgun hunters use adaptive encounter-conditional heuristics, not non-conditional Lévy flights, when searching for prey. Using a theoretically derived Bayesian model, we estimate changes in turning-angle and search velocity as a function of encounters with prey at lagged time-steps, and find that: 1) hunters increase average turning-angle in response to encounters, producing a more tortuous search of patches of higher prey density, but adopt more efficient uni-directional, inter-patch movement after failing to encounter prey over a sufficient period of time; and, 2) hunters reduce search velocity in response to encounters, causing them to spend more of their search time in patches with demonstrably higher prey density. These results illustrate the importance of using event-level data to contrast encounter-conditional, area-restricted search and Lévy flights in explaining the search behavior of humans and other organisms.

## Introduction

Active search for prey is energetically costly [1], so understanding how foragers optimize random search has been central to foraging theory [2–4]. A theoretical and applied literature centered on the Lévy flight foraging hypothesis [4–6] has suggested that foragers should search for randomly distributed prey items using approximations to Lévy flights, since Lévy flights are frequently more efficient than Brownian movement at encountering sparse, randomly distributed prey. The key to the efficiency of Lévy search is that it balances search across spatial scales [4, 7]. Some studies have successfully tested this hypothesis in humans (e.g., [8, 9]) and non-human animals (e.g., [10, 11]), while others have either failed to find evidence of Lévy movement [12] or documented different patterns of search in humans [13, 14] and other organisms [11, 15]. Issues with measurement, however, have sometimes complicated tests of the underlying ideas [11, 16].

In contrast to the initial work on the Lévy flight foraging hypothesis, more recent theoretical work on intermittent and two-stage search [17, 18] examines how prey encounters [19, 20] can trigger area-restricted search behavior [21] in which both step-size and turning-angle [22] are modified. This work demonstrates that foragers can use simple heuristics and the information provided by prey encounters to strategically adapt search direction and velocity, outperforming non-conditional Lévy movement by minimizing search time [17], increasing encounter rate, and/or decreasing risk of starvation [20]. Nevertheless, these encounter-conditional search heuristics can produce gross movement patterns similar in structure to Lévy movement. If prey are distributed in a patchy manner, then Lévy-like movement can emerge from the interaction of the predator’s search heuristics with the patchy distribution of prey in the environment [23–25]. In order to test whether Lévy-like movement patterns of foragers arise as a function of fixed, cognitive-behavioral mechanisms for Lévy movement or as a result of the interaction of encounter-conditional search heuristics and patchily distributed prey, it is necessary to explicitly model step-size (the distance traveled between consecutive GPS—Global Positioning System—points) and turning-angle (the difference in heading direction between consecutive GPS points) as a function of prey encounters at lagged time-steps.

Due to the difficulty of collecting concurrent GPS data on forager movement and prey encounters, there has been limited empirical research on the event-level effects of prey encounters on search mode in humans. Some previous studies have therefor used simulated environments (e.g., [13]) to study search dynamics. In recent years, however, a small number of ground-truthed data-sets on foraging behavior linked to indicators of prey presence have been produced (e.g., [26]). In the non-human arena, researchers have been able to test for effects of prey encounters on the movement dynamics of free-ranging animals through the use of clever research designs, for instance by using GPS tracks of long-range foraging by albatrosses coupled with data on prey capture gained by monitoring stomach temperature with a swallowed recording device [27], or by using the GPS tracks of northern gannets linked to dive events inferred from temperature and pressure recorders [28].

More often, however, the influences of prey density on forager movement patterns have been inferred at broad spatial scales [10, 11]. For example, a key study of the movement patterns of 14 marine predators [11] suggests that, while Lévy search patterns dominate in less productive ecosystems, foragers switch to Brownian search patterns in more productive ecosystems. The authors view these findings as being consistent with the Lévy flight foraging hypothesis contingent on environmental abundance [3, 11]. However, unmeasured confounding variables between the two ecological settings—sparse off-shelf, and productive shelf, habitats—limit inferences about the individual- or encounter-level factors driving predator search dynamics.

In previous work on the topic, there has been a limited connection between the formal theoretical models of optimal search dynamics used to predict behavior and the statistical tools used to analyze empirical GPS data. While it is common to analyze only the distributional properties of step-size and turning-angle, another method of analysis involves using a generative theoretical model [20] to link encounters with prey-items at a given time-step to changes in step-size and turning-angle in subsequent time-steps. Such models demonstrate that simple encounter-conditional search heuristics can lead to movement patterns resembling both Lévy flights and Brownian motion, as well as intermediate forms of search. As such, event-level analysis of encounter-annotated GPS tracks using such a generative model is needed to effectively test the quantitative predictions that can disentangle these two explanations for Lévy-like movement patterns.

Here we use encounter-annotated GPS tracking data to show that two indigenous Colombian blowgun hunters use adaptive encounter-conditional heuristics and not non-conditional Lévy flights when searching for prey. Specifically, we use a theoretically derived Bayesian model to estimate changes in turning-angle and search velocity as a function of encounters with prey at lagged time-steps. We find that: 1) hunters increase turning-angle in response to encounters, producing a more tortuous search of patches of higher prey density, but adopt more efficient uni-directional, inter-patch movement after failing to encounter prey over a sufficient period of time; and, 2) hunters reduce step-size (i.e., search velocity) in response to encounters, causing them to spend a greater fraction their search time in patches with demonstrably higher prey density. These results illustrate the importance of using event-level data and encounter-conditional modeling to contrast adaptive, encounter-conditional search heuristics and non-conditional Lévy flights in explaining the foraging behavior of humans and other animals.

## Methods

Previous research has argued that we must take the influence of prey density [11] or, more specifically, prey encounters [19] into account when analyzing forager movement patterns. Accordingly, we analyze encounter-annotated GPS movement data collected during 18.7 hours of focal follows of two Colombian Emberá-Chamí [29] blowgun hunters using a generative time-series model. We first present our data and then outline the modeling approach.

### Data collection

As a demonstration of the feasibility of our methods, focal follows with two experienced Emberá-Chamí hunters were conducted in the spring of 2017 in Risaralda, Colombia. These individuals hunted using blowguns and darts, sometimes dipped in poison derived from frogs (*Phyllobates*). Hunts were carried out in primary and secondary rain-forest, on the frontier between the mountainous Risaralda department and the lowland Chocó department.

The first author (CTR) accompanied a single hunter on each hunt of 2–4.5 hours in duration, recording GPS positions using a Bad Elf Pro+ receiver unit (BE-GPS-2300). CTR maintained a position 1–2 meters from the hunter during slow movement and rest, and 2–4 meters during more rapid movement. This positioning kept observer and hunter consistently within the error range of the GPS unit. GPS positions were recorded every 10 seconds and the data were analyzed at this resolution. Table 1 provides descriptive statistics of each hunt. We analyze only those GPS data-points collected during active hunting; on three hunts, the GPS logger was stopped when the hunters reached a distant camp to rest—the return phases of these trips were purely locomotive, and are not included in the analyses. Informed consent was obtained from participants prior to data collection. All field protocols were approved by the Max Planck Institute for Evolutionary Anthropology, Department of Human Behavior, Ecology and Culture, who deemed the study exempt from IRB review. Further approvals are described in the S1 Appendix.

**Table 1:** Descriptive statistics of each hunting trip. For each hunt, we describe the number of GPS data points collected, the distance traveled (km), the time duration (hrs), the average speed (km/hr), the change in elevation between minimal and maximal points on the hunt (m), the number of GPS points with prey encounters, the number of shots fired, the number of shots which hit their target, and the total number of prey items recovered.

Hunt	GPS Points	Dist. (km)	Time (hr)	Avg. Speed (km/hr)	Elevation Change (m)	Encountered	Shots	Hits	Recovered
1	1551	4.40	4.31	1.02	322.9	11	3	3	0
2	1639	3.76	4.55	0.83	179.4	29	9	3	2
3	711	1.97	1.98	1.00	56.8	0	0	0	0
4	1021	1.92	2.84	0.68	255.6	26	7	3	0
5	1055	2.22	2.93	0.76	334.4	18	4	2	0
6	754	1.94	2.09	0.93	246.0	14	6	2	0
Sum	6731	16.21	18.70	5.22	1395.1	98	29	13	2
Mean	1122	2.70	3.12	0.87	232.5	16	5	2	0

**Fig 1:**
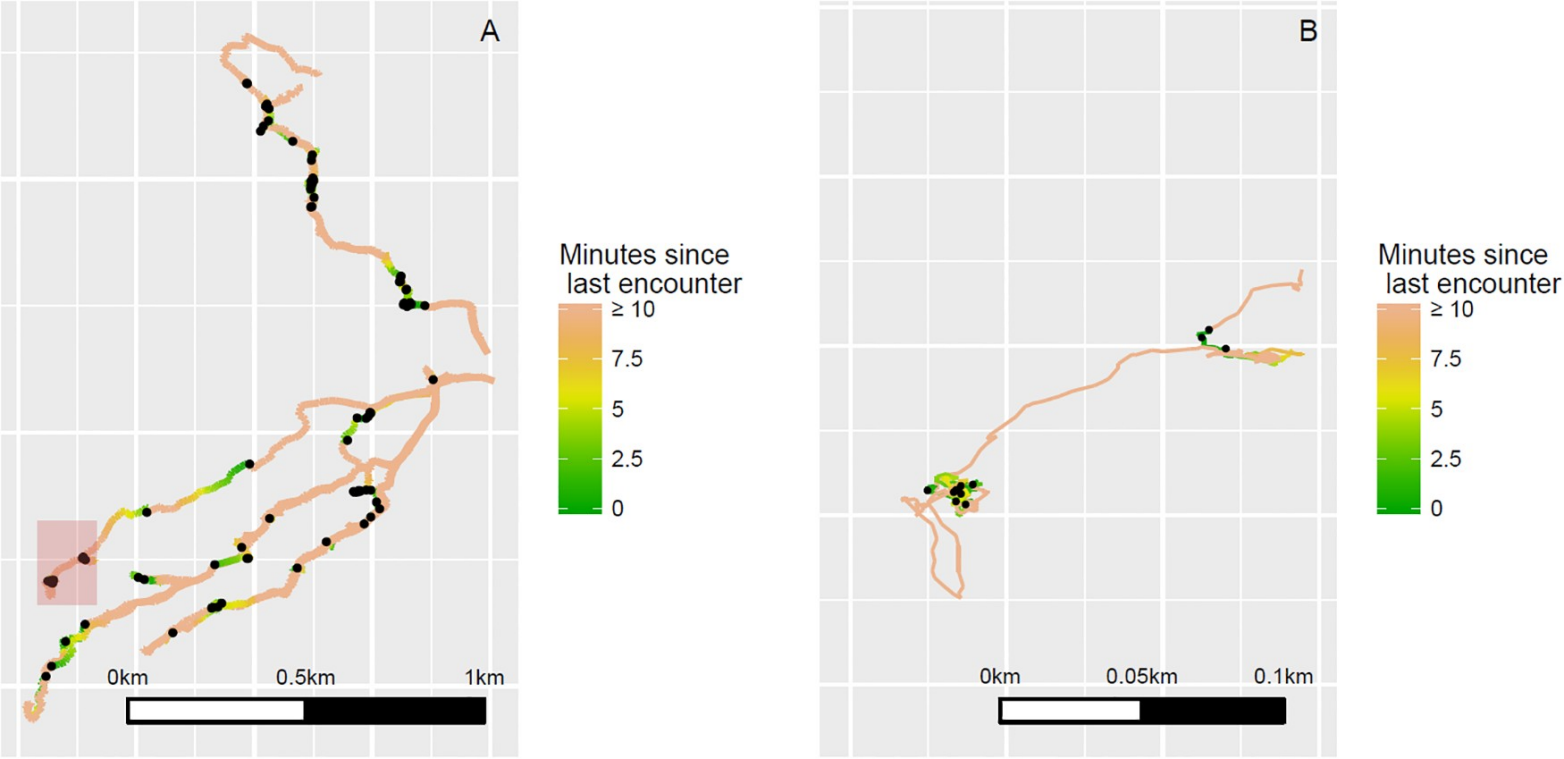
Focal follow GPS recordings of Emberá-Chamí blowgun hunters, spring 2017. Frame A plots smoothed, low-resolution paths for all hunts in the data set. At this large scale, hunts appear fairly linear. Frame B plots a higher-resolution image of the red-shaded area from Frame A. At this scale, it becomes evident that prey encounters (black points plotted on each respective path) are associated with changes in search style. Note that the paths are colored by time since last encounter with prey. The orange regions, representing search periods at least 10 minutes post-encounter, are mostly linear. The green regions, representing search periods shortly after an encounter, are more tortuous (Frame B).

In total, the database is comprised of 6,731 GPS data-points divided into 6 separate hunting trips. Five hunts were conducted with the most experienced hunter in the small community, and an additional hunt was conducted with another experienced hunter from the same community. Annotated points of interest were recorded on the GPS unit for each of the 98 prey encounters that occurred over the course of search. The left frame of Fig 1 plots all search paths and encounters; the right frame plots a higher-resolution section of the foraging path on a single trip.

### Modeling GPS data

Guided by recent mathematical models [19, 20] and theory on area-restricted search [21, 27], we predict that foragers searching for spatially auto-correlated or patchily distributed prey items—e.g., social prey or otherwise solitary prey congregating on common environmental attractors like fruiting trees—should search using two adaptive encounter-conditional heuristics to determine movement:

1. If the forager has not recently encountered a prey item, then the forager is unlikely to be in a localized area of elevated prey density (i.e., a patch) and should thus reduce turning angles in order to adopt a more linear direction of travel. This minimizes the probability of backtracking into areas already established as having a low probability of yield. Alternatively, if the forager has recently encountered a prey item, then the forager is more likely to be in an area of relatively high local prey density and should minimize the probability of leaving it by increasing turning-angle [27, 30].
2. If the forager has not recently encountered a prey item, then the forager is likely to be between patches of elevated prey density, and should take larger step-sizes (i.e., move at a higher velocity). Outside of a patch, it does not pay to search in great detail. However, once in a rewarding patch, as evidenced by a recent encounter with prey, it is best to reduce step-size to minimize the probability of leaving the patch or spooking prey with rapid movement [21, 27].

In order to test these predictions, we analyze encounter-annotated GPS tracking data. The data represent a forager’s search path as a sequence of discrete points in space, (*x*_[*t*]_, *y*_[*t*]_), with a constant separation of ten seconds. These data are easily transformed to a more theoretically relevant form via Cartesian-to-polar mapping [31]. We can parameterize the data so that $r_{\left[t\right]}\in R^{+}$ gives the linear distance between points (*x*_[*t*]_, *y*_[*t*]_) and (*x*_[*t*−1]_, *y*_[*t*−1]_), and *θ*_[*t*]_ ∈ (−*π*, *π*) gives the corresponding heading-angle:

$$\begin{array}{c} r_{\left[t\right]}=\sqrt{{\left(x_{\left[t\right]}-x_{\left[t-1\right]}\right)}^{2}+{\left(y_{\left[t\right]}-y_{\left[t-1\right]}\right)}^{2}} \end{array}$$ (1)

$$\begin{array}{c} \theta_{\left[t\right]}=\arctan^{⋆}|\frac{\left(y_{\left[t\right]}-y_{\left[t-1\right]}\right)}{\left(x_{\left[t\right]}-x_{\left[t-1\right]}\right)}| \end{array}$$ (2)

where the arctan^⋆^ function is the standard arctan function after adjusting the angle for the quadrant of the point in Cartesian space [31]. Then, we transform heading-angle (an absolute direction) into turning-angle, by considering the difference in heading-angle between time steps. The unit-scaled turning-angle, *δ*_[*t*]_, is:

$$\begin{array}{c} \delta_{\left[t\right]}=\frac{\Delta (\theta_{\left[t\right]},\theta_{\left[t-1\right]})}{\pi } \end{array}$$ (3)

where the Δ(*a*, *b*) function returns the minimum of: |*a* − *b*| and 2*π* − |*a* − *b*|, since a 90 degree right turn is the same as a 270 degree left turn, for example. Division by *π* radians yields a value on the unit interval.

Since turning-angle is a unit-constrained variable, we can most effectively model its distribution using a Beta regression model (see [32] for a formal justification):

$$\begin{array}{c} \delta_{\left[t\right]}∼\text{Beta}\left(\mu_{\left[t\right]}\nu ,\left(1-\mu_{\left[t\right]}\right)\nu \right) \end{array}$$ (4)

The mean of the Beta distribution at time *t* is then given by *μ*_[*t*]_:

$$\begin{array}{c} \mu_{\left[t\right]}={\text{logit}}^{-1}(\psi_{\left[0\right]}+\sum_{s=1}^{S}\psi_{\left[s\right]}E_{\left[t-s\right]}) \end{array}$$ (5)

and the dispersion of the distribution for a fixed *μ* is controlled by $\nu \in R^{+}$. *E*_[*t*]_ is an indicator variable of whether a prey item was encountered at time-step *t*, $\psi \in R^{S+1}$ is a vector of unknown parameters estimating the effects of encounters on turning-angle over *S* time-step lags, and logit^−1^ is the inverse logit function. This equation allows us to estimate how lagged encounters affect turning-angle in the current time-step.

The Lévy flight foraging hypothesis assumes that turning-angle should be distributed uniformly—a special case of the Beta distribution with parameters: *ψ*_[0]_ = 0, *ψ*_[*s*]_ = 0, and *ν* = 2. The adaptive encounter-conditional search model predicts that: *ψ*_[*s*]_ > 0, with the other parameters having no specific *a priori* values [20].

Regarding the distribution of step-sizes, we use a log-normal model. Information theoretic model comparison of step-size in Lévy-like movement in humans [33] has shown that the log-normal distribution outperforms other commonly used distributions.

Specifically, we model the distribution of step-sizes as:

$$\begin{array}{c} r_{\left[t\right]}∼\text{Log-Normal}\left(\eta_{\left[t\right]},\omega \right) \end{array}$$ (6)

where the mean of the log of the step-size at time *t* is given by *η*_[*t*]_:

$$\begin{array}{c} \eta_{\left[t\right]}=(\phi_{\left[0\right]}+\sum_{s=1}^{S}\phi_{\left[s\right]}E_{\left[t-s\right]}) \end{array}$$ (7)

and the dispersion of the distribution of the log of step-size for a fixed *η* is controlled by $\omega \in R^{+}$. *E*_[*t*]_ is the same indicator of whether a prey item was encountered at time-step *t*, and $\phi \in R^{S+1}$ is a vector of unknown parameters estimating the effects of encounters on step-size over *S* time-step lags. This equation allows us to estimate how lagged encounters affect step-size in the current time-step.

The Lévy flight foraging hypothesis predicts that: *ϕ*_[*s*]_ = 0 and that *ω* is large enough to produce a heavy-tailed distribution [20]. The adaptive encounter-conditional search model predicts that: *ϕ*_[*s*]_ < 0, with the other parameters having no specific *a priori* values [20].

The raw data, model code, and additional methodological details are available in the S1 Workflow; the data set and code will also be maintained at www.github.com/ctross/emberasearch. Analysis of data was conducted using R [34] and Rstan [35].

## Results

**Fig 2:**
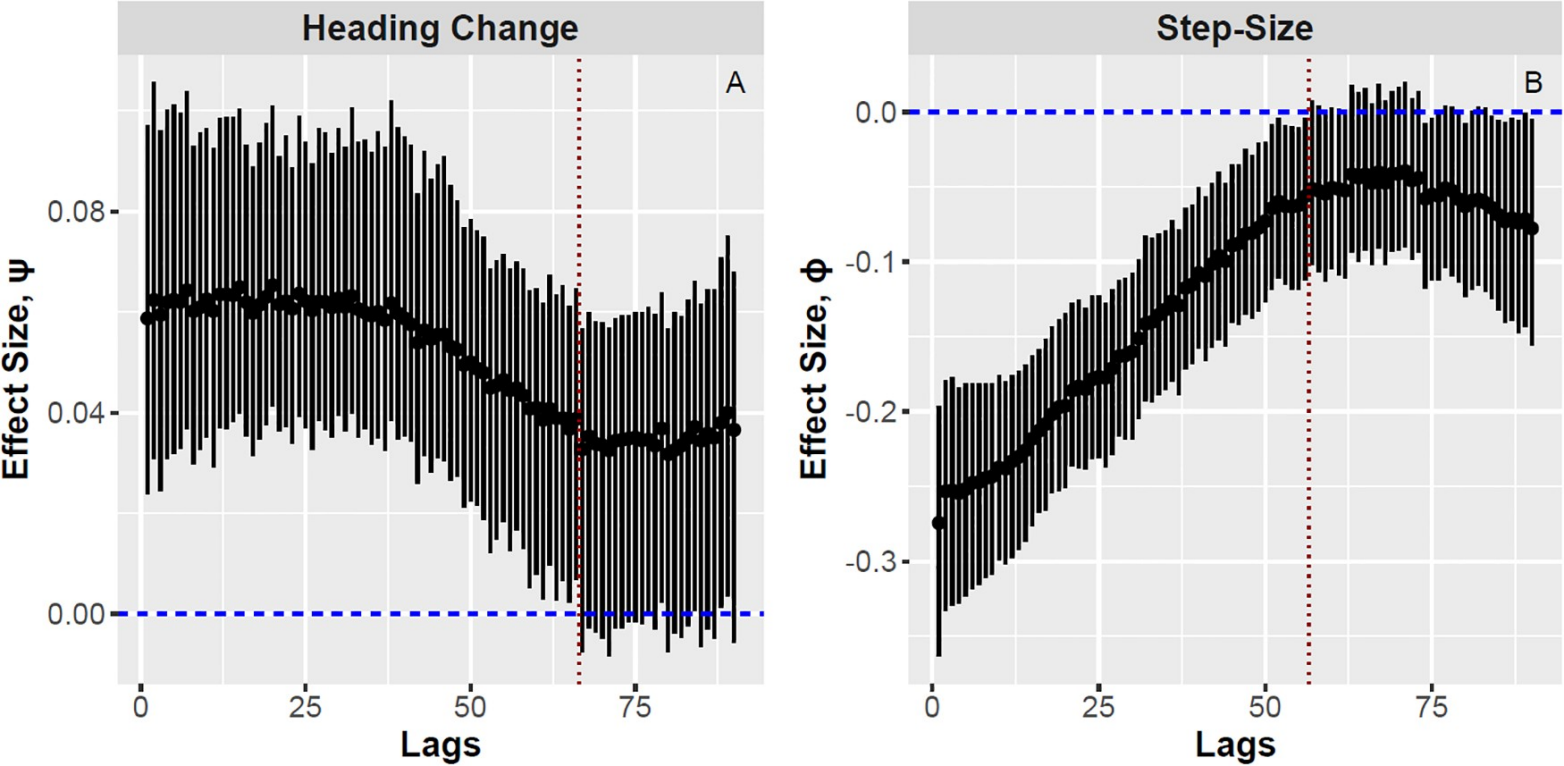
Posterior distributions for the effects of prey encounters on turning-angle and step-size. Both frames depict medians and 90% credible intervals. Frame A plots *ψ*_[*s*]_—the lagged effects of encounters on turning-angle, and Frame B plots *ϕ*_[*s*]_—the lagged effects of encounters on step-size, for lags *s* ∈ {1, …, 90}. The horizontal blue lines represent an effect size of zero. We note significant effects of lagged encounters on both turning-angle (positive) and step-size (negative), with effects lasting about 66 time-steps (11 minutes) for turning-angle change, and about 56 time steps (9 minutes) for step-size. The vertical red lines indicate when the confidence regions on the parameters begin to cross zero, the value of no effect.

Although our preliminary sample is small in terms of hunts and hunters, it is large in terms of encounters (*n* = 98) and observation points (*n* = 6, 731). As such, we were able to document statistically reliable evidence that these blowgun hunters used adaptive encounter-conditional heuristics to guide their search behavior on these hunts. The left frame of Fig 2 plots the posterior distributions (medians and 90% credibility intervals) of *ψ*_[*s*]_, which give the lagged effects of encounters on turning-angle for each lag *s* ∈ {1, …, 90}. The right frame plots the corresponding estimates of *ϕ*_[*s*]_, which give the lagged effects of encounters on step-size. Since each lag, *s*, represents a 10 second interval, the graphs depict lagged effects lasting up to 15 minutes post-encounter. The regression estimates of *ψ*_[*s*]_ remain significantly greater than 0 for *s* < 66, indicating that prey encounters are associated with increased turning changes for about 11 minutes. The regression estimates of *ϕ*_[*s*]_ remain significantly less than 0 for *s* < 56, indicating that encounters are associated with decreased hunter velocity for about 9 minutes.

Our results are robust to two checks. We use an AR-1 model (a 1-lag auto-regression, see [36]) to control for potential temporal auto-correlation in turning-angle and step-size. And, we model turning-angle and step-size using *ψ*_[*s*]_ and *ϕ*_[*s*]_ parameters that are unique to two types of encounters: those in which prey were shot and hit, and those in which prey were not. We make this assessment because the behavior of the forager after the first type of encounter might reflect the behavioral pattern of item recovery rather than continued search. Under both controls, our qualitative findings remain unchanged; see S1 Appendix for details.

**Fig 3:**
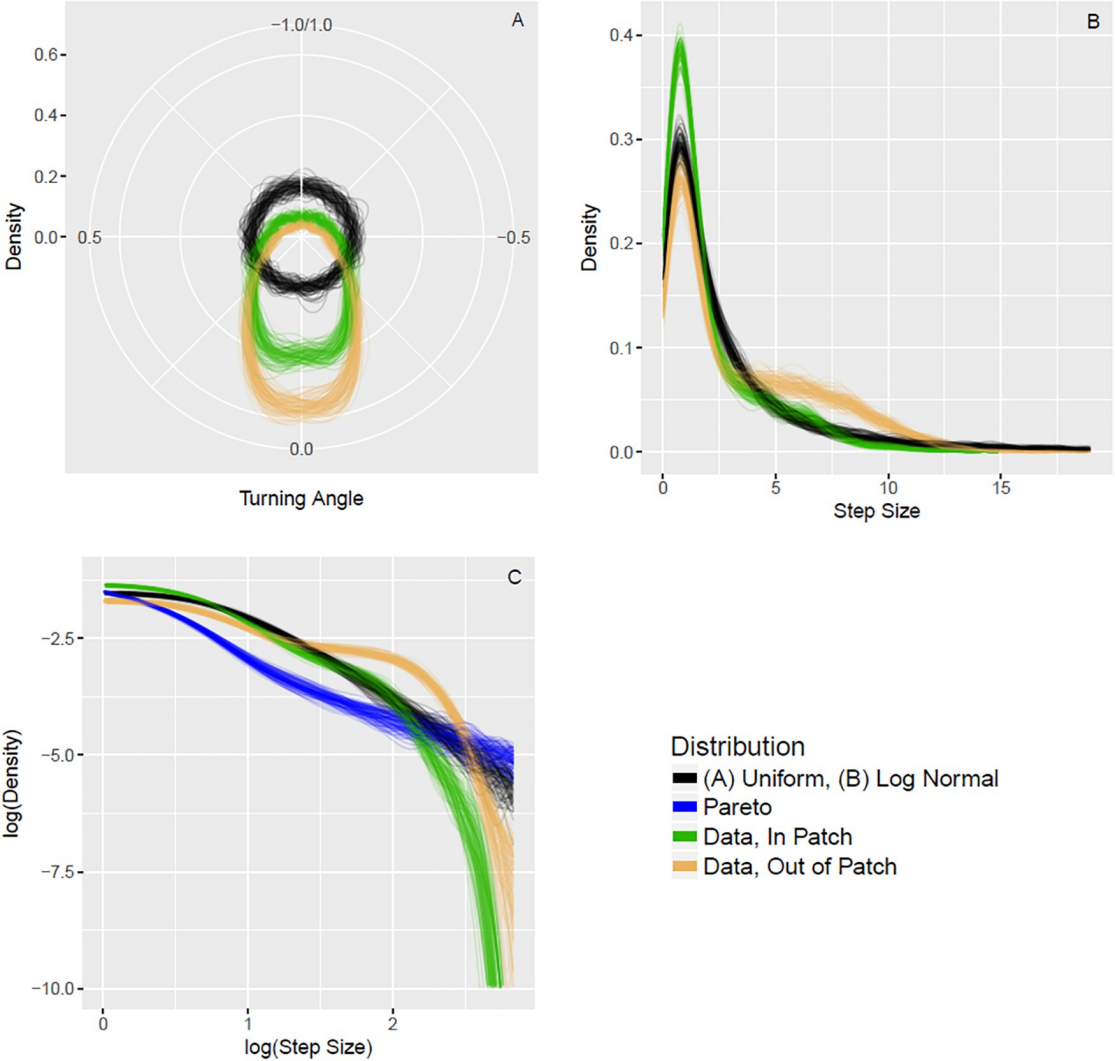
Kernel densities (with boot-strap resampling) for turning-angle (Frame A, plotted on polar coordinates) and step-size (Frame B). Frame C is equivalent to Frame B on a log-log scale. The green curves plot the data distributions when the foragers were in a patch (specifically, for time-steps when the forager had an encounter within the previous 50 time-steps). The orange curves plot the data distributions when the foragers were not in a patch. In Frame A, the black curves illustrate the uniform turning-angle distribution assumed in the Lévy flight foraging hypothesis. In Frames B and C, the black curves illustrate the best-fitting log-normal step-size distribution. In Frame C, the blue curves are from the best-fitting truncated Pareto distribution. The turning-angle distributions are measured in units of *π* radians, and the step-size distributions are in units of meters. The step-size measure reflects the linear distance between adjacent GPS points in meters in each 10 second time interval.

Our blowgun hunting data are inconsistent with non-conditional Lévy search patterns. The empirical turning-angle data depart from the uniform distribution assumed under the Lévy flight foraging hypothesis and the empirical step-size distribution lacks the expected power-law-like tail (see Fig 3, and Watanabe-Akaike information criterion [37, 38] in Table 2). Instead, we observe a high density near a step-size of zero, which arises from the hunters waiting or walking silently in areas near to where prey items were recently spotted, and a moderate range of step-sizes for the inter-patch phases of the hunts.

**Table 2:** Watanabe-Akaike information criterion [37, 38] comparisons for the distribution of step-size. Standard and truncated folded-normal, log-normal, and Pareto distributions were considered. The truncated log-normal distribution, with parameters: *μ* = 0.64 (CI95: 0.60, 0.68), *σ* = 1.39 (CI95: 1.36, 1.42), and *τ* = 18.73 (CI95: 18.70, 18.83), outperforms other models in the set. Note here that the *μ* and *σ* parameters of the log-normal distribution define, respectively, the mean and standard deviation of the variable’s natural logarithm; the *τ* parameter defines the variable’s upper truncation point.

	WAIC	Δ-WAIC	Weight
Truncated Log Normal	28186.80	0.00	1.00
Truncated Folded Normal	28237.10	50.40	0.00
Folded Normal	28246.00	59.20	0.00
Log Normal	28652.10	465.40	0.00
Truncated Pareto	36944.60	8757.80	0.00
Pareto	46423.40	18236.60	0.00

The elevated density on small turning angles arises from the preference of the hunters to use ridge-line paths and stream beds to travel quickly and directionally, reflecting underlying environmental influences on search dynamics. Once they encounter a prey item, however, they tend to slow down (i.e., reduce step-size) and engage in a more careful exploration of their surroundings through use of larger turning-angles. Sometimes they sit and wait for out-of-range prey items to wander into range, and sometimes they begin a slow and quiet attempt to maneuver themselves into a position from which they can shoot the prey item. In most cases, this slower hunting style persists after the harvest or escape of the prey item that triggered the slow-down. Frequently, when a prey item is encountered by a hunter, that prey item is residing in a tree that is fruiting and the hunters prefer to stay close in hopes that other prey items will be located in, or arrive to, the same location.

In our predictions, we proposed that certain heuristics can lead to increased encounter-rates when inside of patches. This has been demonstrated in simulated environments [19, 20]. If for our purposes here, we define a patch as those data-points occurring within 50 time-steps of a prey encounter, inferred on the basis of the estimates in Fig 2, then we can fit some simple regression models to generate slope coefficients, *β*, measuring key effects. First, we note that turning-angle is reliably greater when inside a ‘patch’ (*β* = 0.08, CI90: 0.07, 0.09) and step-size is significantly less when inside a ‘patch’ (*β* = −1.48, CI90: −1.61, −1.36). Additionally, hunters increase their probability of an encounter at time-step *t* as a function of increased turning-angle (*β* = 0.76, CI90: 0.12, 1.41) and decreased step-size (*β* = −0.13, CI90: −0.20, −0.05) at time *t* − 1. The focal hunters adopt slower and more non-linear movement while in ‘patches’ and this slower and more non-linear, in-patch movement leads to increased encounter rates.

## Discussion

Previous theoretical work has shown that Lévy flights can be efficient methods of search for random and not necessarily uniformly distributed prey items. However, non-conditional Lévy search does not allow foragers to make contingent use of the information they gain during a hunt, information that can further improve search efficiency [19, 20, 39–41]. If prey items show any degree of spatial correlation, then hunters can use encounters to infer the relative local density of prey and then choose movement parameters—turning-angle and step-size—to increase search efficiency. The birds and squirrels that Emberá-Chamí hunters target appear to become clustered in space as a result of the asynchronous fruiting of their forest food sources. Emberá-Chamí hunters, aware of this fact, use initial prey encounters to adaptively change their search mode as predicted by theory.

Our results are consistent with previous observations of area-restricted search in humans and non-human animals (e.g., [13, 27, 28, 42–44]), and theoretical analysis of intermittent search strategies, which adaptively mix periods of locomotion and inspection [18]. The human hunters followed in this study appear to update their movement mode as a function of recent encounters with prey items. This result is consistent with the idea that they use adaptive heuristics rather than non-conditional Lévy flights to guide their movement. The parameters controlling the effects of encounters at lagged time-steps on the search mode of the hunters estimated in the Bayesian model reliably exclude the numerical value of zero expected under a strict formulation of the Lévy flight foraging hypothesis. Nevertheless, the GPS tracks recorded during focal follows of these hunters do show some elements of Lévy-like movement; specifically, we observe long tracks of directional movement interspersed with periods of area-restricted search or sitting-and-waiting. Our event-level method of data analysis has allowed us to demonstrate that these periods of area-restricted search are triggered by prey encounters. These results thus provide evidence that apparent Lévy-like movement in humans might be explainable as a by-product of the interaction of some simple search heuristics and the distribution of prey items in the environment, as has been suggested in prior work on non-human foragers [23, 25].

We note a few important qualifications about our results. First, our sample of hunts and hunters is limited. Although we have a large sample of prey encounters with which we can estimate encounter-conditional changes in search mode, only a larger sample of hunters will allow us to test for inter-individual variation in the relationship between encounters and foraging mode. Second, we see route fidelity across several hunts, indicating that hunters use some information from their environment to guide their hunts. As noted previously, these hunters prefer to follow ridge-line paths and stream beds when traveling quickly between patches rather than clearing paths through more difficult terrain. While theoretical models of Lévy flights and other search modes typically assume an unconstrained environment in which a hunter can move, human and non-human hunters must select paths on the basis of real-world factors like vegetation density, ease of movement, slope, and their own conspicuousness. Most previous empirical tests of the Lévy flight foraging hypothesis have been conducted on animals whose movement is arguably less constrained by the environment (e.g., pelagic predators [27, 28] or canopy primates [23]). The fact that the hunters in our study did not appear to hunt using non-conditional Lévy flights could be due, in part, to the fact that Lévy flights are inefficient in the context of the specific constraints imposed by the environment. Finally, our results are based on human hunting patterns and humans have long-term memory of habitat structure and prey encounters that may exceed that of other animals. For example, some snails have been shown to move in Lévy-like walks even in the absence of encounters with food items [24]. Their Lévy-like movement seems to be inherent rather than habitat driven [24]. The possibility remains that different animals might use different heuristics during search. The analytical methods developed here will allow for wide, cross-species comparisons to test such possibilities.

Previous studies contrasting Lévy movement and encounter-conditional, area-restricted search in humans have relied on use of simulated environments [13]. In the current analysis, we rely on focal follows of hunters, with an anthropologist recording annotations on a Bad Elf GPS unit and separate vocal recording device. Newer GPS units feature integrated, one-touch GPS and vocal recording, opening the possibility for foragers to annotate their own hunts. While it is quite laborious to simultaneously record the GPS tracks of foragers in natural environments and annotate their prey encounters, our findings demonstrate the potentially wide-ranging importance of studying human and other foragers and their prey as a coupled system using event-level data and analysis.

## Supporting information

S1 Appendix Additional methodological details and results of robustness checks. (PDF)

S1 Workflow Data and code. (ZIP)
