# Supplementary material for: Evidence for encounter-conditional, area-restricted search in a preliminary study of Colombian blowgun hunters
Source: PLoS One. 2018 Dec 12;13(12):e0207633. doi: 10.1371/journal.pone.0207633 (PMC6291100; doi:10.1371/journal.pone.0207633)
Supplement: S1 Appendix — (PDF) [file pone.0207633.s001.pdf]

Supplementary Materials to: Evidence for encounter-conditional, area-restricted search in a preliminary study of Colombian blowgun hunters

Cody T. Ross[1] and Bruce Winterhalder[2]
[1]*Max Planck Institute for Evolutionary Anthropology. Department of Human Behavior, Ecology and Culture. Deutscher Platz 6. Leipzig, 04103. Germany.*
[2]*University of California, Davis. Department of Anthropology. 1 Shields Ave. Davis, CA 95616. USA.*

1 Full Model Description

In order to test our predictions, we use a theoretical model[1] to analyze encounter-annotated GPS tracking data, which represent a forager's search path as a sequence of discrete points in space, ($x_{[t]}$, $y_{[t]}$), with a constant temporal separation (the time steps here represent 10 second intervals). These data are easily transformed to a more theoretically relevant form via Cartesian-to-polar mapping[2]. We can parameterize the data so that $r_{[t]} \in \mathbb{R}^+$ gives the linear distance between points ($x_{[t]}$, $y_{[t]}$) and ($x_{[t-1]}$, $y_{[t-1]}$), and $\theta_{[t]} \in (-\pi, \pi)$ gives the corresponding heading-angle:

$$r_{[t]} = \sqrt{(x_{[t]} - x_{[t-1]})^2 + (y_{[t]} - y_{[t-1]})^2} \quad (1)$$

$$\theta_{[t]} = \arctan^{\star} \left| \frac{(y_{[t]} - y_{[t-1]})}{(x_{[t]} - x_{[t-1]})} \right| \quad (2)$$

where the $\arctan^{\star}$ function is the standard $\arctan$ function after adjusting the angle for the quadrant of the point in Cartesian space [2]. Then, we transform heading-angle (an absolute direction) into turning-angle, by considering the difference in heading-angle between time steps. The unit-scaled turning-angle, $\delta_{[t]}$, is:

$$\delta_{[t]} = \frac{\Delta(\theta_{[t]}, \theta_{[t-1]})}{\pi} \quad (3)$$

where the $\Delta(a, b)$ function returns the minimum of: $|a - b|$ and $2\pi - |a - b|$, since a 90 degree right turn is the same as a 270 degree left turn, for example. We divide by π radians to yield a value on the unit interval.

Since turning angle is interval constrained, we model its distribution using a Beta regression[3] framework:

$$\delta_{[t]} \sim \text{Beta}(\mu_{[t]}\nu, (1 - \mu_{[t]})\nu) \quad (4)$$

The mean of the Beta distribution at time t is then given by $\mu_{[t]}$:

$$\mu_{[t]} = \text{logit}^{-1} \left(\psi_{[0]} + \sum_{s=1}^{S} \psi_{[s]} E_{[t-s]} \right) \quad (5)$$

and the dispersion of the distribution for a fixed μ is controlled by $\nu \in \mathbb{R}^+$. $E_{[t]}$ is an indicator variable of if a prey item was encountered at time-step t, $\psi \in \mathbb{R}^{S+1}$ is a vector of unknown parameters estimating the effect of encounters on turning angle over S time-step lags, and logit^{-1} is the inverse logit function.

The dispersion parameter ν has a weak, positive-constrained (i.e. truncated between 0 and ∞) prior:

$$\nu \sim \text{Normal}(1, 0.5)T[0, \infty] \quad (6)$$

The intercept parameter $\psi_{[0]}$ has a weak prior:

$$\psi_{[0]} \sim \text{Normal}(0, 5) \quad (7)$$

The parameters $\psi_{[1:S]}$ controlling the effect of lagged encounters on turning angle are partially pooled (locally) using a Gaussian Random Field[5] approach:

$$\psi_{[1:S]} = \Psi + \delta_{[1:S]} \tag{8}$$

where Ψ gives a mean effect and $\delta_{[1:S]}$ are mean-zero offsets from Ψ:

$$\delta_{[1:S]} \sim \text{Multi. Norm. Cholesky}((0, \ldots, 0)', \sigma_\delta L_\delta) \tag{9}$$

σ_δ is a scalar of variance, and L_δ is a factor from the Cholesky decomposition of the correlation matrix, ρ_δ, which is in turn defined for $i \neq j$ as:

$$\rho_{\delta_{[i,j]}} = \gamma_\delta \exp\left(-\kappa_\delta \frac{(i-j)^2}{S^2}\right) \tag{10}$$

and as $\rho_{\delta_{[i,j]}} = 1$, for $i = j$.

All hyperparameters are given weakly informative priors:

$$\Psi \sim \text{Normal}(0, 2.5) \tag{11}$$

$$\sigma_\delta \sim \text{Normal}(0, 5)T[0, \infty] \tag{12}$$

$$\gamma_\delta \sim \text{Beta}(12, 2) \tag{13}$$

$$\kappa_\delta \sim \text{Normal}(0, 5)T[0, \infty] \tag{14}$$

We use a similar model to estimate the effects of encounters on the distribution of step-size. The outcomes are modeled as:

$$r_{[t]} \sim \text{Log-Normal}(\eta_{[t]}, \omega) \tag{15}$$

where the probability density function for the log-normal distribution[4] is:

$$\text{Log-Normal}(x|\alpha, \beta) = \frac{1}{\sqrt{2\pi}\beta}\frac{1}{x}\exp\left(-\frac{1}{2}(\frac{\log x - \alpha}{\beta})^2\right) \tag{16}$$

and α and β are the location and scale parameters, respectively, of the distribution of $\log(x)$.

The mean of the log of the step-size at time t is given by $\eta_{[t]}$:

$$\eta_{[t]} = \left(\phi_{[0]} + \sum_{s=1}^{S} \phi_{[s]} E_{[t-s]}\right) \tag{17}$$

and the dispersion of the distribution of the log of step-size for a fixed η is controlled by $\omega \in \mathbb{R}^+$. $E_{[t]}$ is the same indicator of if a prey item was encountered at time-step t, and $\phi \in \mathbb{R}^{S+1}$ is a vector of unknown parameters estimating the effect of encounters on step-size over S time-step lags.

The dispersion parameter ω has a weak, positive-constrained prior:

$$\omega \sim \text{Cauchy}(0, 1)T[0, \infty] \tag{18}$$

The intercept parameter $\phi_{[0]}$ has a weak prior:

$$\phi_{[0]} \sim \text{Normal}(0, 5) \tag{19}$$

The parameters $\phi_{[1:S]}$ controlling the effect of lagged encounters on step-size are partially pooled (locally) using a Gaussian Random Field approach:

$$\phi_{[1:S]} = \Phi + \xi_{[1:S]} \tag{20}$$

where Φ gives a mean effect and $\xi_{[1:S]}$ are mean zero offsets from Φ:

$$\xi_{[1:S]} \sim \text{Multi. Norm. Cholesky}((0, \ldots, 0)', \sigma_\xi L_\xi) \tag{21}$$

σ_ξ is a scalar of variance, and L_ξ is a factor from the Cholesky decomposition of the correlation matrix, ρ_ξ, which is in turn defined for $i \neq j$ as:

$$\rho_{\xi_{[i,j]}} = \gamma_\xi \exp\left(-\kappa_\xi \frac{(i-j)^2}{S^2}\right) \tag{22}$$

and as $\rho_{\xi_{[i,j]}} = 1$, for $i = j$.

All hyperparameters are given weakly informative priors:

$$\Phi \sim \text{Normal}(0, 2.5) \tag{23}$$

$$\sigma_\xi \sim \text{Normal}(0, 5)T[0, \infty] \tag{24}$$

$$\gamma_\xi \sim \text{Beta}(12, 2) \tag{25}$$

$$\kappa_\xi \sim \text{Normal}(0, 5)T[0, \infty] \tag{26}$$

2 Robustness Checks: An AR-1 Model

Since the turning angle and step-size data derived from GPS points might potentially show strong temporal auto-correlation, we also use an AR-1 (a 1-lag auto-regression) style model to analyze our data. All aspects of the model remain constant, with the exception of Eqs. 5 and 17, which now read as:

$$\mu_{[t]} = \text{logit}^{-1}\left(\psi_{[0]} + \zeta\delta_{[t-1]} + \sum_{s=1}^{S} \psi_{[s]} E_{[t-s]}\right) \tag{27}$$

and:

$$\eta_{[t]} = \left(\phi_{[0]} + \chi r_{[t-1]} + \sum_{s=1}^{S} \phi_{[s]} E_{[t-s]}\right) \tag{28}$$

with ζ and χ controlling the effects of the outcomes at one lag on the current outcomes. They have priors:

$$\zeta \sim \text{Normal}(0, 5) \tag{29}$$

$$\chi \sim \text{Normal}(0, 5) \tag{30}$$

We find significantly positive values for ζ=1.69 (95PCI: 1.55, 1.81) and χ=0.036 (95PCI: 0.037, 0.038), indicating that both turning-angle and step-size are auto-correlated in time. However, our main findings are qualitatively robust to this control. See Figure 1, which demonstrates that even after controlling for temporal auto-correlation, encounters continue to affect search mode as presented in the main text.

[Figure 1 about here.]

3 Robustness Checks: Encounter Types

Since the turning-angle and step-size data derived from GPS points might potentially show unique patterns after encounters in which prey were shot and hit (prey recovery) and encounters in which prey were not hit (continued search), we fit an additional model with independent effects for each kind of encounter. All aspects of the model remain constant, with the exception of Eqs. 5 and 17, which now read as:

$$\mu_{[t]} = \text{logit}^{-1}(\psi_{[0]} + \sum_{s=1}^{S} (\psi_{[s]} Z_{[t-s]} + \hat{\psi}_{[s]} H_{[t-s]})) \tag{31}$$

and:

$$\eta_{[t]} = \phi_0 + \sum_{s=1}^{S} (\phi_{[s]} Z_{[t-s]} + \hat{\phi}_{[s]} H_{[t-s]}) \tag{32}$$

with $\hat{\psi}_{[s]}$ and $\hat{\phi}_{[s]}$ controlling the lagged effects of encounters in which prey were hit with an arrow, H, on the current outcomes. They have the same priors as $\psi_{[s]}$ and $\phi_{[s]}$. In this model, $\psi_{[s]}$ and $\phi_{[s]}$ control the lagged effects of encounters in which prey were not hit with an arrow, Z, on the current outcomes.

We find that the results of the main model are robust to this control as well. See Figure 2.

[Figure 2 about here.]

4 Problematic Data Points

Due to limited sensitivity of the GPS receiver, a total of 151 out of 6,731 (i.e. $\sim$2.2%) otherwise usable data-points had a distance estimate of exactly 0 units. This value is problematic since the log-normal distribution has no support on the point of zero. To fix this issue, we treat these values as truncated data points and we impute each of them a single time from a random uniform distribution. Each realization is constrained to fall between 0 and the minimum possible detection distance of 0.1 units.

Is cases where the distance estimate between points is zero, turning-angle is undefined. As such, in cases where the distance estimate equals zero, we also impute a random realization of turning angle. In this case, we treat these values as missing data points and impute each of them a single time from the best approximating Beta distribution to the empirical distribution of turning-angle.

More robust full Bayesian imputation is another possible approach here, but would significantly increase the run time of our already computationally intensive models. By imputing a fixed realization for each of these truncated and missing data points, we are introducing noise unconditional on our predictors, which should, if anything, lead to underestimation of effects relative to the full Bayesian approach. This works against our hypotheses, which nonetheless remain supported.

5 Approvals

The first author obtained a TP-7 visa, required to conduct research in Colombia, prior to data collection. Informed consent was obtained from each hunter and the community leader prior to data collection. Because of limited literacy rates at the study site, informed consent was obtained verbally. The verbal consent was not recorded. All field protocols were approved by the Max Planck Institute for Evolutionary Anthropology, Department of Human Behavior, Ecology and Culture.

1. Ross, C., Pacheco-Cobos, L. & Winterhalder, B. A general model of forager search: Adaptive encounter-conditional heuristics outperform Lévy flights in the search for patchily distributed prey. *Journal of Theoretical Biology* (2018). URL http://www.sciencedirect.com/science/article/pii/S0022519318303552.

2. Weisstein, E. W. Polar coordinates (2002). URL http://mathworld.wolfram.com/PolarCoordinates.html.

3. Ferrari, S. & Cribari-Neto, F. Beta regression for modelling rates and proportions. *Journal of Applied Statistics* **31**, 799–815 (2004).

4. Stan Development Team. Stan modeling language users guide and reference manual, version 2.18.0 (2018). URL http://mc-stan.org/.

5. McElreath, R. *Statistical rethinking: A Bayesian course with examples in R and Stan*, vol. 122 (CRC Press, 2016).

List of Figures

1 Posterior distributions for turning-angle and step-size in the AR-1 model. Both frames depict medians and 90% credibility intervals. The left frame plots $\psi_{[s]}$, the lagged effects of encounters on turning-angle, and the right frame plots $\phi_{[s]}$, the lagged effects of encounters on step-size, for lags $s \in \{1, \ldots, 90\}$. We note significant effects of lagged encounters on both turning-angle and step-size, with effects lasting about 66 time-steps (11 minutes) for turning-angle, and about 30 time steps (5 minutes) for step-size, as indicated by the vertical red bars. These results are qualitatively similar to the main analysis, with the effects being the same in direction, though slightly shorter in duration and smaller in magnitude. . . 6

2 Posterior distributions for turning-angle and step-size in the model with two encounter types. Both frames depict medians and 90% credibility intervals. The left frame of each subfigure plots $\psi_{[s]}$, the lagged effects of encounters on turning-angle, and the right frame of each subfigure plots $\phi_{[s]}$, the lagged effects of encounters on step-size, for lags $s \in \{1, \ldots, 90\}$. In the case of encounters not resulting in prey being stuck with arrows, we note significant effects of lagged encounters on both turning-angle and step-size, with effects lasting about 60 time-steps (10 minutes) for turning-angle, and about 50 time steps (8.33 minutes) for step-size, as indicated by the vertical red bars. These effects are of comparable direction, magnitude, and duration to the effects presented in the main text. In the case of prey being struck by arrows, we see stronger effects on turning angle, but more moderate reductions in step-size. This difference occurs because prey recovery typically involves an active search for a struck prey item, but encounters not leading to prey items being hit typically generate slower and less conspicuous movement, as the forager continues the hunt. 7

Figure 1: Posterior distributions for turning-angle and step-size in the AR-1 model. Both frames depict medians and 90% credibility intervals. The left frame plots $\psi_{[s]}$, the lagged effects of encounters on turning-angle, and the right frame plots $\phi_{[s]}$, the lagged effects of encounters on step-size, for lags $s \in \{1, \ldots, 90\}$. We note significant effects of lagged encounters on both turning-angle and step-size, with effects lasting about 66 time-steps (11 minutes) for turning-angle, and about 30 time steps (5 minutes) for step-size, as indicated by the vertical red bars. These results are qualitatively similar to the main analysis, with the effects being the same in direction, though slightly shorter in duration and smaller in magnitude.

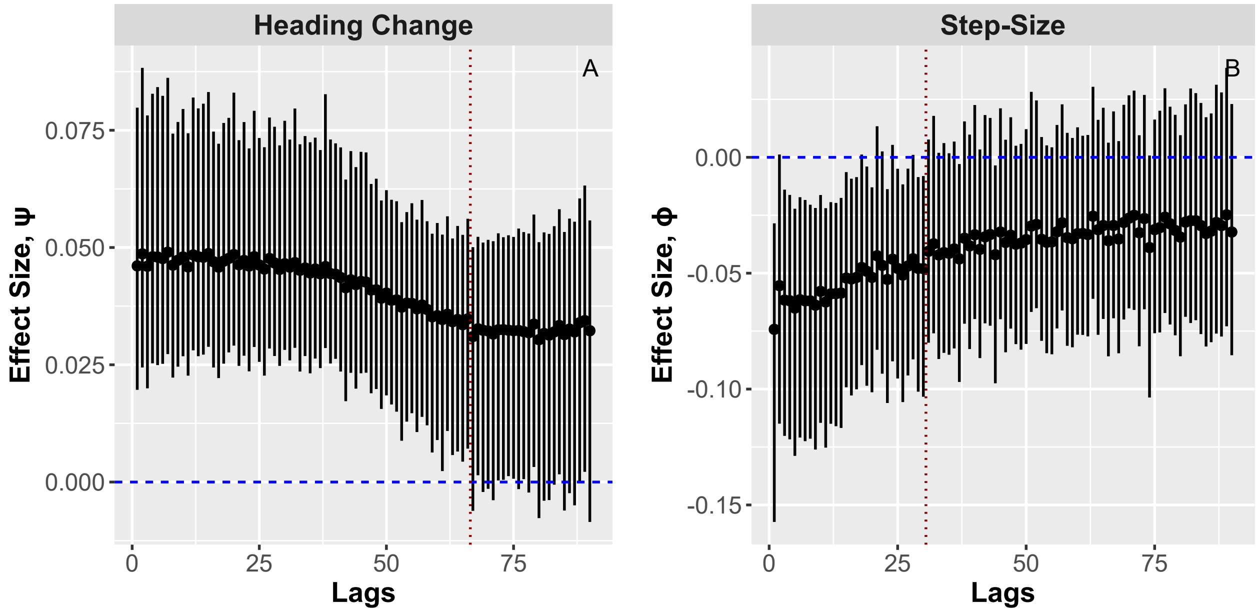

Figure 2: Posterior distributions for turning-angle and step-size in the model with two encounter types. Both frames depict medians and 90% credibility intervals. The left frame of each subfigure plots $\psi_{[s]}$, the lagged effects of encounters on turning-angle, and the right frame of each subfigure plots $\phi_{[s]}$, the lagged effects of encounters on step-size, for lags $s \in \{1, \ldots, 90\}$. In the case of encounters not resulting in prey being stuck with arrows, we note significant effects of lagged encounters on both turning-angle and step-size, with effects lasting about 60 time-steps (10 minutes) for turning-angle, and about 50 time steps (8.33 minutes) for step-size, as indicated by the vertical red bars. These effects are of comparable direction, magnitude, and duration to the effects presented in the main text. In the case of prey being struck by arrows, we see stronger effects on turning angle, but more moderate reductions in step-size. This difference occurs because prey recovery typically involves an active search for a struck prey item, but encounters not leading to prey items being hit typically generate slower and less conspicuous movement, as the forager continues the hunt.

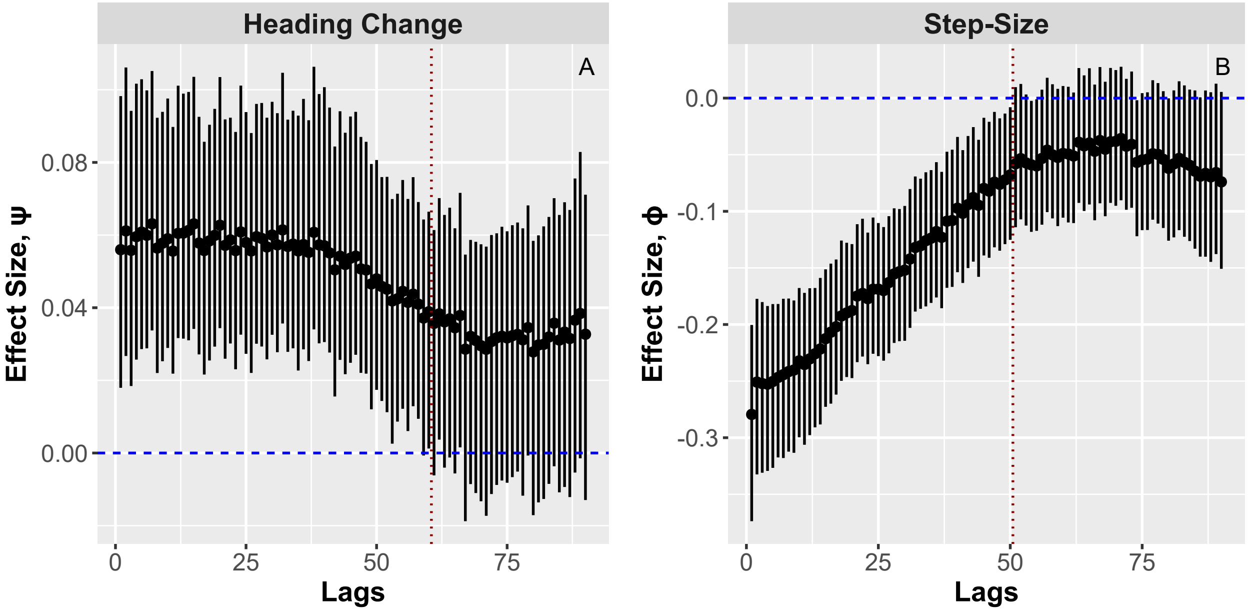

(a) Effects of encounters *not resulting* in prey items being hit with an arrow.

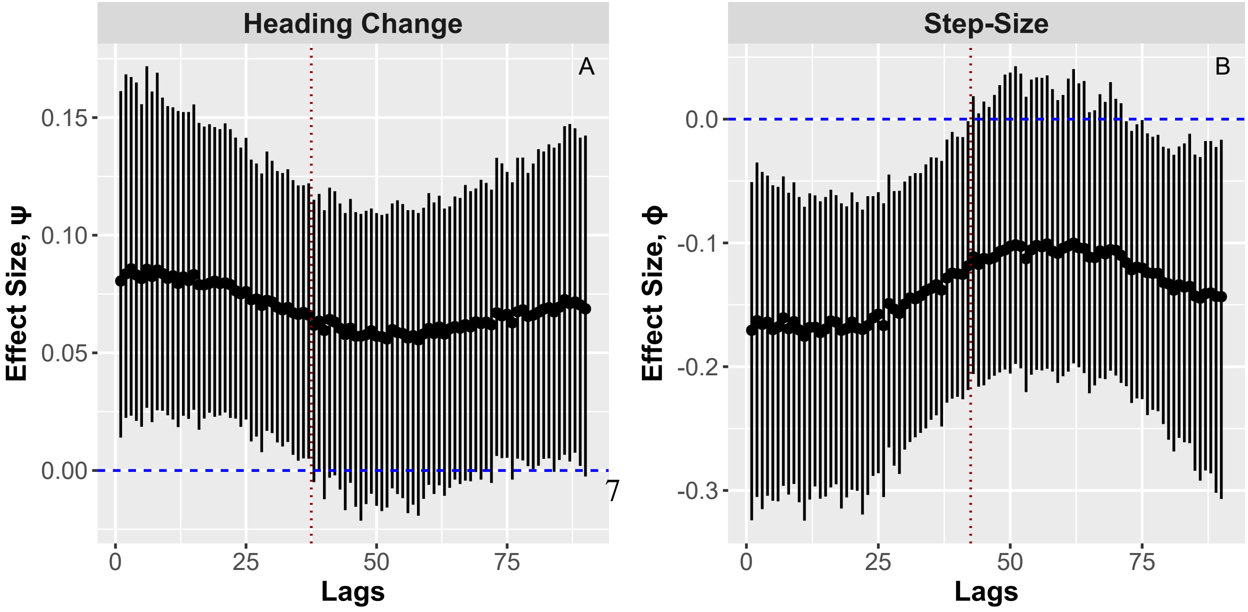

(b) Effects of encounters *resulting* in prey items being hit with an arrow.
